# The association between non-communicable diseases and COVID-19 severity and mortality among infected hospitalized healthcare workers in 29 countries: a cohort study

**DOI:** 10.12688/f1000research.150838.1

**Published:** 2024-06-12

**Authors:** Yusuf Sheku Tejan, Jacklyne Ashubwe, Mher Beglaryan, Shermarke Hassan, Sartie Kenneh, Francis Moses, Abdulai Tejan Jalloh, Fassou Mathias Grovogui, Ibrahima Kaba, Sia Morenike Tengbe, Mustapha Kabba, Mamud Idriss Kamara, Santigie Sesay, Jonta Kamara, Jerry-Jonas Mbasha, Pryanka Relan, Innocent Nuwagira, Ibrahim Franklyn Kamara

**Affiliations:** 1 Ministry of Health, 4th Floor, Youyi Building, Freetown, Sierra Leone; 2 Medwise Solutions Consultancy, Nairobi, P.O. Box 2356 00202, Kenya; 3 Tuberculosis Research and Prevention Centre, Yerevan, 0014, Armenia; 4 Infectious Diseases Data Observatory, University of Oxford, Oxford, UK; 5 Nuffield Department of Medicine, Center for Tropical Medicine and Global Health, University of Oxford, Oxford, UK; 6 Africa Center of Excellence for the Prevention and Control of Communicable Diseases, University Gamal Abdel Nasser, Conakry, Guinea; 7 Multidisciplinary Adolescent and Youth Review Board, Second Lancet Commission on Adolescent Health and Wellbeing, Toronto, Ontario, Canada; 8 World Health Organization, Africa Regional Office, Brazzaville, Congo; 9 World Health Organization, Headquarters, Geneva, Switzerland; 10 World Health Organization Country Office, 21 A-B Kingharman Road, Freetown, Sierra Leone

**Keywords:** COVID-19, Healthcare workers, Noncommunicable diseases, Mortality, Disease severity, SORT IT

## Abstract

**Background:**

Due to occupational exposure, healthcare workers (HCWs) have a higher risk of Coronavirus Disease 2019(COVID-19) infection than the general population. Non-communicable diseases (NCDs) may increase the risk of COVID-19-related morbidity and mortality among HCWs, potentially reducing the available health workforce. We examined the association between NCDs and COVID-19 disease severity and mortality among infected HCWs.

**Methods:**

This cohort study used data from the International Severe Acute Respiratory and Emerging Infections Consortium (ISARIC) database. HCWs hospitalized between January 2020 and January 2023 due to clinically suspected or laboratory-confirmed COVID-19 were eligible for inclusion. Variables collected included demographic data, comorbidities, and hospitalization outcomes. Descriptive statistics were reported using mean/standard deviation (SD), median/interquartile range (IQR), or frequencies and proportions. For each NCD, the relative risk of death, adjusted for age and sex, was calculated using log-binomial regression as well as the population-attributable fraction.

**Results:**

There were 17,502 HCWs, 95.7% of whom had a confirmed COVID-19 diagnosis. The majority were female (66.5%) and the mean age (SD) was 49.8 (14.3) years. Roughly, half (51.42%) of HCWs had no comorbidities, 29.28% had one comorbidity, 14.68% had 2 comorbidities and <5% had ≥3 comorbidities. The most common comorbidities were diabetes mellitus (49.40%) and cardiovascular disease (36.90%). Approximately one-fifth of the HCWs had severe COVID-19 (16.95%) and 10.68% of the HCWs with COVID-19 died. Being ≥45 years old, male gender, smoking, obesity, and certain NCDs increased the risk of COVID-19 severity and mortality. Obesity and diabetes mellitus were the leading risk factors in terms of the population-attributable risk for COVID-19 severity (6.89%) and mortality (36.00%) respectively.

**Conclusions:**

Many HCWs with COVID-19 had one or more NCDs. Obesity and diabetes mellitus increased COVID-19 severity and mortality risk. Reducing the prevalence of obesity and diabetes mellitus would yield the biggest reduction in COVID-19-related morbidity and mortality among HCWs.

## Introduction

The world was grappling with the triple burden of communicable and non-communicable diseases (NCDs) and injuries, amplified by climate change effects, when it was struck by the Coronavirus Disease (COVID-19) pandemic caused by the Severe Acute Respiratory Syndrome Coronavirus- 2 (SARS-CoV-2) virus. ^1^^–^^5^ In December 2019, the first case of COVID-19 was detected in Wuhan China. Within a short period the disease had spread to all regions of the world which compelled the World Health Organization (WHO) to declare it a public health emergency of international concern (PHEIC) in January 2020 and characterize it as a pandemic in March 2020 ( WHO - COVID-19). This novel virus continued to evolve and spread with emerging strains accounting for increased risk of transmission and different degrees of disease severity within the community and in healthcare settings. ^6^

Healthcare workers (HCWs) bore the brunt of the pandemic through occupational exposure as they cared for COVID-19 patients. ^7^ There is a growing body of evidence showing that HCWs were at higher risk of contracting the virus compared to the general population, as they cared for COVID-19 patients. ^8^^,^^9^ The resultant negative physical and mental impacts of the disease on HCWs are well-documented. ^10^^,^^11^

HCWs serving in low-and-middle-income countries (LMICs), whose health systems are plagued by resource constraints, were more likely to be adversely affected due to gaps in infection prevention and control (IPC) mechanisms. ^11^^–^^13^ A recent study conducted in Sierra Leone documented a high (29%) COVID-19 secondary infection rate among HCWs at three regional hospitals. ^14^ Additionally, two other studies reported that HCWs experienced increased psychological stress including anxiety, isolation, fear, and being overwhelmed particularly in the first three months of the pandemic. ^15^^,^^16^

Furthermore, a recent systematic review documented geographical variations in COVID-19-related case fatality rates among HCWs. ^17^ The highest mortality was seen in the Eastern Mediterranean (5.7%) followed by Southeast Asia (3.1%), Africa (1.2%), and Europe (0.6%). ^17^ These differences in mortality rates might have been due to several factors including age, sex, comorbidities, and health system structures. ^18^

A country-based study in Egypt indicated that chronic diseases and home-based management were predictors of COVID-19 severity among HCWs. ^19^ Evidence from a systematic review concurred with these findings that NCDs potentiated the severity of COVID-19 manifestations in the general population. ^20^ These studies highlight the relationship between highly transmissible communicable diseases like COVID-19 and NCDs.

Globally, WHO reports that NCDs cause 41 million deaths annually. ^21^ The most common NCDs are cardiovascular diseases including hypertension, diabetes mellitus, chronic obstructive pulmonary diseases, and cancers. ^21^ LMICs, previously plagued primarily by infectious diseases, are now seeing an upward trend in NCDs and their attendant disabilities. A recent systematic review indicated that sub-Saharan African countries had seen an increase of 67% in disability-adjusted life years (DALYs) attributable to NCDs between 1990 and 2017. ^22^

These trends have been associated with an increase in modifiable behavioural factors such as tobacco and harmful alcohol use, low consumption of fruits and vegetables, high intake of salts and sugar, low-fibre diets, obesity, and low physical activity. These behavioural factors are observed both in the general population and among HCWs. ^23^ Several country-specific studies have documented evidence of risk factors for the development of NCDs among HCWs. A study conducted in Brazil estimated a high overall NCD prevalence (30%) among nurses. ^24^ Another study conducted across several provinces in Zimbabwe documented that half (50%) of HCWs had at least one NCD with hypertension (36%) being the most common. ^25^

NCDs, when present as comorbidities, increase the risk of poor treatment outcomes and mortality. Evidence has shown that patients with pre-existing NCDs who were infected with COVID-19 had an increased risk of severe disease and mortality. ^8^^,^^26^ A systematic review documented a two-fold increase in severity and a three-fold mortality risk in COVID-19 patients with underlying diabetes mellitus. ^26^

NCDs may increase the risk of COVID-19-related morbidity and mortality among HCWs, potentially reducing the available health workforce. Few studies have been conducted on the effects of NCDs on COVID-19 among HCWs, who have a higher exposure to the disease. Furthermore, no analysis has been conducted on the association between COVID-19 severity and mortality and risk posed by NCDs such as hypertension, diabetes mellitus, or other NCDs among HCWs from an multinational dataset.

To address this knowledge gap, we conducted a multi-country study among HCWs hospitalized for COVID-19 to (i) describe their demographic attributes and prevalence of NCDs and other risk factors, (ii) examine the association between NCDs and other risk factors and COVID-19 severity and mortality, and (iii) estimate the population-attributable fraction (PAF) of COVID-19 severity and death due to NCDs and other risk factors.

The findings and recommendations from this study may inform policies and strategies for reducing the vulnerability of HCWs in future public health emergencies and promote the resilience of health systems in the face of such shocks.

## Methods

This was a cohort study that used secondary data collected in 29 countries spread across different geographical regions. The study period ran from January 2020 to January 2023. Patients’ records were followed up from hospital admission to exit.

### General setting

Globally, 60 countries distributed across Africa, Europe, North and South America, Australia, and the Arab and South-East Asian regions reported COVID-19 cases in healthcare facilities as part of the International Severe Acute Respiratory and Emerging Infections Consortium (ISARIC) study. Data collection, aggregation, curation, and harmonisation processes of the ISARIC study have been described previously. ^27^

### Specific setting

Twenty-nine countries out of the 60 reporting COVID-19 data into the ISARIC database had reported information on HCWs hospitalized for COVID-19. The reporting countries were Egypt, Gambia, Ghana, Guinea, Libya, Malawi, South Africa, Estonia, Germany, Italy, Ireland, Luxembourg, Poland, Spain, United Kingdom, Argentina, Brazil, Canada, Peru, United States of America, Jordan, Kuwait, Palestine, United Arab Emirates, Australia, Hong Kong, Indonesia, Malaysia, and the Philippines.

### Study population

HCWs who were hospitalized with clinically suspected or laboratory-confirmed COVID-19 from the 29 countries that reported information on HCWs to the ISARIC database from January 2020 to January 2023 were eligible for inclusion.

### Data collection and variables

Participating sites used the ISARIC-WHO case report form to enter data into a Research Electronic Data Capture ( REDCap version 8.11.11, Vanderbilt University, Nashville, TN) database or the local databases before uploading to the Infectious Diseases Data Observatory (IDDO) database ( Open Data Kit is a suitable open access alternative). Centrally collated data were wrangled and mapped to the structure and controlled terminologies of the Study Data Tabulation Model version 1.7, Clinical Data Interchange Standards Consortium, Austin, TX) using TrifactaVR software ( OpenRefine is a suitable open access alternative). The independent variables were patient demographic information and the presence of NCD comorbidities; disease severity and patient outcomes were the dependent variables.

### Operational definitions

Severe COVID-19 was defined as one or more of the following: admission to an intensive care unit (ICU), treatment with invasive mechanical ventilation (IMV), non-invasive ventilation (NIV), high-flow nasal cannulas (HFNC), extra-corporeal membrane oxygenation (ECMO), administration of inotropes and/or vasopressors. Severity was calculated for cases where all the components were not missing. The following comorbidities were assessed; diabetes mellitus (any type), asthma, cardiovascular disease, chronic pulmonary disease (not asthma), rheumatological disorder, chronic kidney disease, malignant neoplasm, chronic neurological disorder, chronic haematological disease, dementia, malnutrition, smoking status and obesity. The data on comorbidities (except obesity) were self-reported. Obesity was defined by the clinicians attending to the patients based on the Body Mass Index of ≥ 30.

### Data analysis

Data was extracted from the IDDO database. ^27^ R code was used to create a dataset for analysis ( R version 4.3.2; R Core Team (2023). R: A Language and Environment for Statistical Computing. R Foundation for Statistical Computing, Vienna, Austria). We used Stata v18.0 [StataCorp. 2023. Stata Statistical Software: Release 18. College Station, TX: StataCorp LLC] to prepare the data for analysis.

To estimate the relative risks (RR) of severity and death, we fitted log-binomial regression models (generalized linear models for the binomial family with the log link) for both severity and death outcomes using glm function of R. We omitted predictors with less than 15 outcome events in their exposed category. We adjusted comorbidities’ RR estimates for age and sex. To that end, we fitted a separate model for each comorbidity where predictors were the comorbidity, age, and sex. A P-value of 0.05 was used as the cut-off for statistical significance.

To estimate the proportion of deaths among HCWs that could be attributed to a specific NCD, we calculated the population attributable fraction (PAF). The PAF (and 95%CI) was calculated for comorbidities that were significant in the adjusted analysis and had RR estimates greater than 1. The R package ‘ graphPAF’ R package version 2.0.0) was used to calculate age-and sex adjusted PAF estimates. ^28^

### Ethics considerations

Ethical approval was obtained via the global IDDO approval from different countries. The WHO Ethics Review Committee (RPC571 and RPC572, Apr 25, 2013) and the local or national Ethics Committees for participating sites approved the Execution of the ISARIC-WHO Clinical Characterization Protocol. Approvals included the South Central—Oxford C Research Ethics Committee for England (Ref. 13/SC/0149), the Scotland A Research Ethics Committee (Ref. 20/SS/0028) for Scotland, and the Human Research Ethics Committee (Medical) at the University of the Witwatersrand in South Africa as part of a national surveillance programme (M160667), which collectively represent most of the data. For patient data collected and used in research, patient consent was waived according to local norms determined by the responsible Ethics Committee. The IDDO governance processes covered the arrangements surrounding the pooling, storage, curation, and sharing of these data.

## Results

### Sociodemographic characteristics of healthcare workers admitted for COVID-19

**Figure 1.**
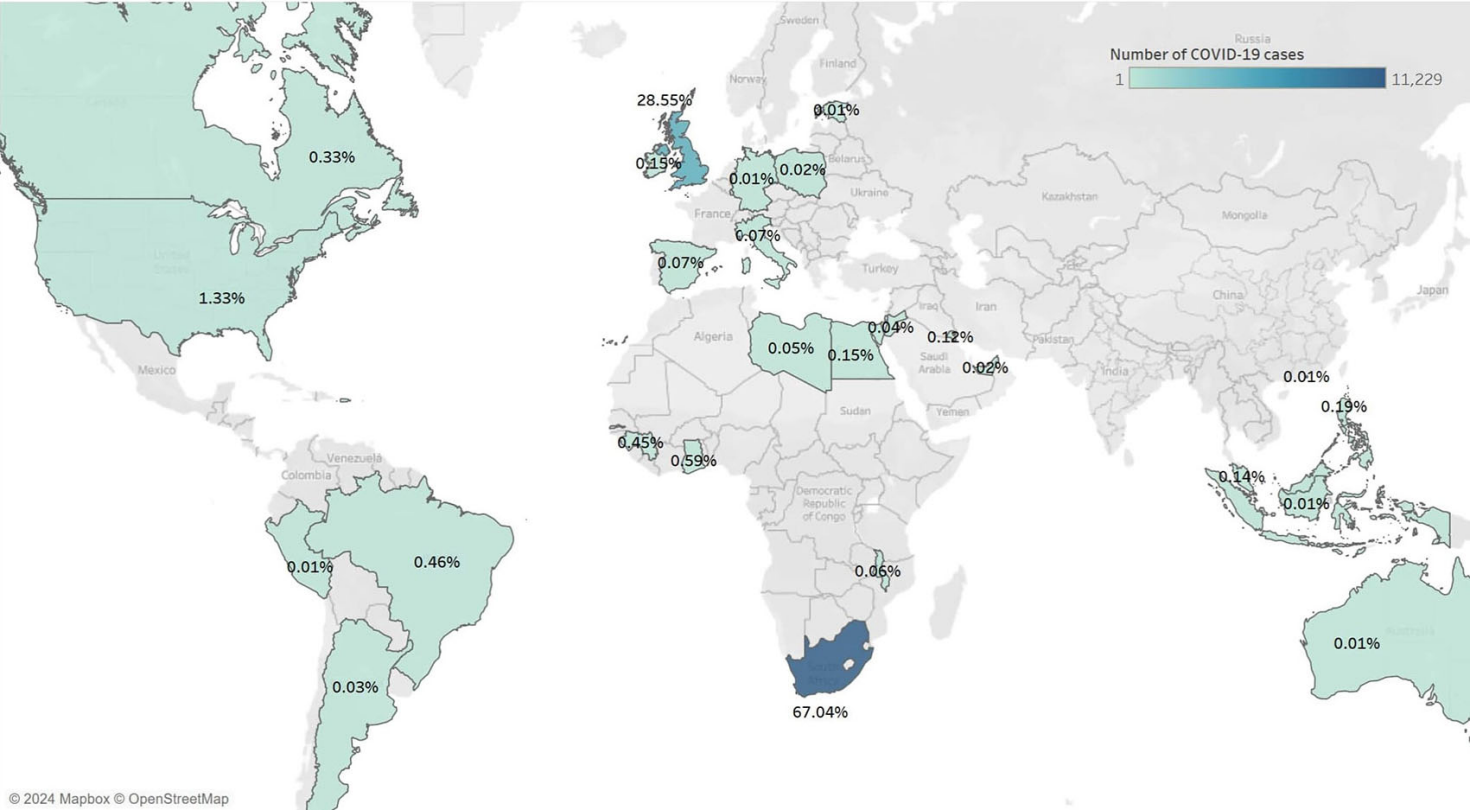
Distribution of healthcare workers hospitalized for COVID-19 from 2020 to 2023 across countries.

A total of 841,640 records of patients admitted with COVID-19 were stored in the IDDO platform. Patients were hospitalized between January 2020 and January 2023. Of these, 17,502 (2.08%) were HCWs. Among these HCWs, a COVID-19 diagnosis was confirmed for 16,750 (95.70%). Of the 16,750 HCWs hospitalized with confirmed COVID-19, the majority (11,130, 66.45%) were females, and the mean age (SD) was 49.78 (14.33). Nearly all HCWs in this study were hospitalized in either South Africa or the United Kingdom (11,229, 67.04% and 4,782, 28.55% respectively) ( Table 1, Figure 1).

**Table 1.** Demographic characteristics and comorbidities and other risk factors among healthcare workers hospitalized for COVID-19 from 2020 to 2023 in 29 countries.

Variables	N	%
Total number of COVID-19 patients	841,640	
Total number of HCWs with COVID-19	16750	1.99
**Age in years**		
15-44	6,032	36.01
45-64	8,295	49.52
≥65	2,275	13.58
Unknown	148	0.88
**Sex**		
Female	11,130	66.45
Male	5,609	33.49
Not specified/Unknown	11	0.07
**Countries**		
South Africa	11,229	67.04
United Kingdom	4,782	28.55
United States of America	222	1.33
Ghana	99	0.59
Brazil	77	0.46
Guinea	76	0.45
Canada	56	0.33
Philippines	31	0.19
Egypt	25	0.15
Ireland	25	0.15
Malaysia	23	0.14
Kuwait	20	0.12
Italy	12	0.07
Spain	11	0.07
Malawi	10	0.06
Libya	8	0.05
Gambia	7	0.04
Jordan	7	0.04
Luxembourg	7	0.04
Argentina	5	0.03
Poland	4	0.02
United Arab Emirates	4	0.02
Estonia	2	0.01
Palestine	2	0.01
Peru	2	0.01
Australia	1	0.01
Germany	1	0.01
Hong Kong	1	0.01
Indonesia	1	0.01
**Number of comorbidities**		
0 Comorbidities	8,613	51.42
1 comorbidity	4,905	29.28
2 comorbidities	2,459	14.68
≥3 comorbidities	773	4.61
**Types of comorbidities** *		
Cardiovascular disease	5,197	36.90
Diabetes mellitus	3,308	49.40
Asthma	1,379	9.80
Chronic pulmonary disease (not asthma)	346	2.50
Rheumatological disorder	293	6.50
Chronic kidney disease	248	1.80
Malignant neoplasm	209	1.50
Chronic neurological disorder	150	3.10
Chronic haematological disease	78	1.70
Dementia	62	1.40
Malnutrition	25	0.50
**Current smoker**		
No	3,815	22.78
Yes	1,056	6.30
Unknown	11,879	70.92
**Obesity**		
No	5,340	31.88
Yes	1,478	8.82
Unknown	9,932	59.30

* Percentages of comorbidities are calculated among non-missing entries.

### Healthcare workers with different comorbidities

At the time of admission, half (8,613, 51.42%) of the HCWs had no comorbidities, more than a quarter (4,905, 29.28%) had one comorbidity, 2,459 (14.68%) had 2 comorbidities, and a small proportion had more than 3 comorbidities (773, 4.61%). The most common comorbidities were diabetes mellitus (3,308, 49.40%), cardiovascular disease (5,197, 36.9%), asthma (1,379, 9.80%), and chronic pulmonary disease (not asthma) (346, 2.50%). About a tenth of the HCWs were obese (1,478, 8.82%) ( Table 1). Percentages of comorbidities were calculated among entries that had no missing data.

### COVID-19 severity and hospitalization outcomes of healthcare workers

2,839 (16.95%) of the HCWs suffered from severe COVID-19 during their period of hospitalization. Less than a quarter (2800, 16.72%) were admitted to the ICU, nearly one quarter (4,012, 23.95%) of the HCWs received oxygen via HFNC and less than one-tenth (1,293, 7.72%) received oxygen via IMV. The proportion of HCWs who died from COVID-19 was 10.68% (1,789) ( Table 2).

**Table 2.** COVID-19 severity and hospitalization outcomes of healthcare workers.

Variables	N	(%)
**Severe COVID-19**		
No	5,414	32.32
Yes	2,839	16.95
Unknown	8,497	50.73
**ICU admission**		
No	13,730	81.97
Yes	2,800	16.72
Unknown	220	1.31
**High-flow oxygen nasal cannulas**		
No	12,120	72.36
Yes	4,012	23.95
Unknown	618	3.69
**Non-invasive ventilation**		
No	7,328	43.75
Yes	1,183	7.06
Unknown	8,239	49.19
**Invasive mechanical ventilation**		
No	15,226	90.90
Yes	1,293	7.72
Unknown	231	1.38
**Extracorporeal membrane oxygenation**		
No	16,421	98.04
Yes	77	0.46
Unknown	252	1.50
**Inotropes, vasopressors**		
No	7,807	46.61
Yes	499	2.98
Unknown	8,444	50.41
**Patient outcomes**		
Survived	14,885	88.87
Death	1,789	10.68
Unknown	76	0.45

### Factors associated with COVID-19 severity and deaths

After adjustment for age and sex, the following factors were significantly associated with severe COVID-19. Age range 45-64 years (RR, 1.91; 95% CI, 1.76-2.07), age ≥ 65 years (RR, 2.02; 95% CI, 1.78-2.27), male gender (RR, 1.52; 95% CI, 1.42-1.62), obesity (aRR, 1.31; 95% CI, 1.21-1.41), smoking (aRR, 1.11; 95% CI, 1.01-1.22), asthma (aRR, 1.24; 95% CI, 1.14-1.35), cardiovascular disease (aRR, 1.14; 95% CI, 1.06-1.23), diabetes mellitus (aRR, 1.00; 95% CI, 0.93-1.08) and chronic pulmonary disease (not asthma) (aRR, 1.22; 95% CI, 1.04-1.41) ( Table 3).

**Table 3.** Relative risks and population attributable fractions (as percents) of COVID-19 severity due to non-communicable diseases among hospitalized health care workers in 29 countries from 2020 to 2023.

		Total (N =7858)		Severe		*Adjusted RR* *^†^* *(95%CI)*		*PAF %* ^††^ *(95%CI)*	
		n	(%)	n	(%)				
**Age in years**									
	15-44	3055	(38.88)	607	(19.87)	Reference			
	45-64	4084	(51.97)	1547	(37.88)	1.91 ***	(1.76-2.07)	NA	
	≥65	602	(7.66)	241	(40.03)	2.02 ***	(1.78-2.27)		
**Sex**									
	Female	5585	(71.07)	1511	(27.05)	Reference			
	Male	2264	(28.81)	931	(41.12)	1.52 ***	(1.42-1.62)	NA	
**Obesity**									
	No	4222	(53.73)	1348	(31.93)	Reference			
	Yes	1371	(17.45)	560	(40.85)	1.31 ***	(1.21-1.41)	6.89	(4.65-9.22)
**Current smoker**									
	No	3199	(40.71)	1025	(32.04)	Reference			
	Yes	985	(12.53)	380	(38.58)	1.11 *	(1.01-1.22)	2.61	(-0.13-5.09)
**Comorbidities**									
Asthma	No	5426	(69.05)	1756	(32.36)	Reference			
	Yes	862	(10.97)	343	(39.79)	1.24 ***	(1.14-1.35)	3.21	(1.75-4.55)
Cardiovascular disease	No	4170	(53.07)	1236	(29.64)	Reference			
	Yes	2275	(28.95)	914	(40.18)	1.14 ***	(1.06-1.23)	5.29	(2.07-8.12)
Diabetes mellitus	No	3220	(40.98)	1240	(38.51)	Reference			
	Yes	1392	(17.71)	583	(41.88)	1.00	(0.93-1.08)	0.08	(-2.59-2.91)
Chronic haematological disease	No	4032	(51.31)	1641	(40.70)	Reference			
	Yes	71	(0.90)	28	(39.44)	0.94	(0.67-1.22)	NA	
Chronic kidney disease	No	6015	(76.55)	2019	(33.57)	Reference			
	Yes	184	(2.34)	60	(32.61)	0.82	(0.65-1.01)	NA	
Malignant neoplasm	No	6011	(76.50)	2022	(33.64)	Reference			
	Yes	160	(2.04)	52	(32.50)	0.84	(0.66-1.04)	NA	
Chronic neurological disorder	No	3975	(50.59)	1626	(40.91)	Reference			
	Yes	132	(1.68)	45	(34.09)	0.81	(0.63-1.01)	NA	
Chronic pulmonary disease (not asthma)	No	5982	(76.13)	1984	(33.17)	Reference			
	Yes	212	(2.70)	97	(45.75)	1.22 **	(1.04-1.41)	0.84	(0.01-1.51)
Rheumatological disorder	No	3826	(48.69)	1546	(40.41)	Reference			
	Yes	281	(3.58)	123	(43.77)	1.02	(0.89-1.17)	0.17	(-0.84-1.1)

NA: Not applicable.

^†^ RR for age and sex are not adjusted.

^††^ Population attributable fraction as percent.

* p-value < 0.05.

** p-value < 0.01.

*** p-value < 0.001.

After adjustment for age and sex, the following factors were significantly associated with mortality from COVID-19. Age range 45-64 years (RR, 3.42; 95% CI, 2.95-3.97), age ≥ 65 years (RR, 8.45; 95% CI, 7.28-9.85), male gender (RR, 1.73; 95% CI, 1.59-1.89), obesity (aRR, 1.34; 95% CI, 1.13-1.57), smoking (aRR, 1.09; 95% CI, 0.88-1.36), dementia (aRR, 1.33; 95% CI, 0.87-1.89), diabetes mellitus (aRR, 2.00; 95% CI, 1.73-2.33), chronic haematological disease (aRR, 1.48; 95% CI, 0.80-2.29), cardiovascular disease (aRR, 1.32; 95% CI, 1.20-1.44), chronic kidney disease (CKD) (aRR, 1.25; 95% CI, 0.98-1.54), malignant neoplasm (aRR, 1.34; 95% CI, 1.04-1.68), chronic neurological disorder (aRR, 1.07; 95% CI, 0.66-1.58), chronic pulmonary disease (not asthma) (aRR, 1.21; 95% CI, 0.97-1.46) and rheumatological disorders (aRR, 1.39; 95% CI, 1.01-1.84) ( Table 4).

**Table 4.** Relative risks and population attributable fractions (as percents) of COVID-19 death due to non-communicable diseases among hospitalized health care workers in 29 countries from 2020 to 2023.

Risks		Total (N =16674)		Death		*Adjusted RR* ^†^ *(95%CI)*		*PAF %* ^††^ *(95%CI)*	
		n	(%)	n	(%)				
**Age in years**									
	15-44	6002	(36.00)	201	(3.35)	Reference			
	45-64	8271	(49.60)	946	(11.44)	3.42 ***	(2.95-3.97)		
	>=65	2258	(13.54)	639	(28.30)	8.45 ***	(7.28-9.85)		
**Sex**									
	Female	11080	(66.45)	956	(8.63)	Reference			
	Male	5583	(33.48)	833	(14.92)	1.73 ***	(1.59-1.89)		
**Obesity**									
	No	5306	(31.82)	458	(8.63)	Reference			
	Yes	1473	(8.83)	160	(10.86)	1.34 ***	(1.13-1.57)	6.41	(2.74-10.5)
**Current smoker**									
	No	3797	(22.77)	244	(6.43)	Reference			
	Yes	1043	(6.26)	100	(9.59)	1.09	(0.88-1.36)	2.53	(-4-9.44)
**Comorbidities**									
Asthma	No	12578	(75.43)	1509	(12.00)	Reference			
	Yes	1371	(8.22)	114	(8.32)	0.78 **	(0.65-0.93)		
Cardiovascular disease	No	8856	(53.11)	775	(8.75)	Reference			
	Yes	5173	(31.02)	884	(17.09)	1.32 ***	(1.20-1.44)	12.70	(8.68-16.5)
Dementia	No	4425	(26.54)	324	(7.32)	Reference			
	Yes	61	(0.37)	19	(31.15)	1.33	(0.87-1.89)	1.32	(-0.60-2.92)
Diabetes mellitus	No	3366	(20.19)	224	(6.65)	Reference			
	Yes	3292	(19.74)	587	(17.83)	2.00 ***	(1.73-2.33)	36.00	(28.8-44.5)
Chronic kidney disease	No	13608	(81.61)	1564	(11.49)	Reference			
	Yes	246	(1.48)	55	(22.36)	1.25	(0.98-1.54)	0.67	(0.18-1.49)
Malignant neoplasm	No	13617	(81.67)	1574	(11.56)	Reference			
	Yes	209	(1.25)	47	(22.49)	1.34 *	(1.04-1.68)	0.71	(0.06-1.28)
Chronic neurological disorder	No	4602	(27.60)	338	(7.34)	Reference			
	Yes	149	(0.89)	17	(11.41)	1.07	(0.66-1.58)	0.30	(-1.81-2.27)
Chronic pulmonary disease (not asthma)	No	13509	(81.02)	1553	(11.50)	Reference			
	Yes	342	(2.05)	69	(20.18)	1.21	(0.97-1.46)	0.72	(-0.24-1.48)
Rheumatological disorder	No	4172	(25.02)	304	(7.29)	Reference			
	Yes	290	(1.74)	38	(13.10)	1.39 *	(1.01-1.84)	3.23	(0.19-6.3)

NA: Not applicable.

^†^ RR for age and sex are not adjusted.

^††^ Population attributable fraction as percent.

* p-value < 0.05.

** p-value < 0.01.

*** p-value < 0.001.

### Population attributable fraction (PAF)

Obesity (PAF, 6.89%; 95% CI, 4.65-9.22) and cardiovascular disease (PAF,5.29%; 95% CI, 2.07-8.12) were found to have a relatively higher population-attributable risk for COVID-19 severity compared to the other factors ( Table 3). Moreover, diabetes mellitus had the highest population-attributable risk for the mortality associated with COVID-19 (PAF, 36.00%; 95% CI, 28.8-44.5) ( Table 4). The PAF for COVID-19 mortality associated with cardiovascular disease was also high, calculated at 12.70% (95% CI, 8.68-16.5).

Under the assumption of a causal relationship between the exposure and, severity and mortality outcomes, we estimated that approximately 6.89% of severe COVID-19 cases and 36.00% of COVID-19-related deaths could potentially be averted if obesity and diabetes mellitus respectively were eliminated as risk factors.

## Discussion

Our study is the most comprehensive multi-country study to evaluate the association between NCDs and COVID-19 severity and deaths. The findings add to the global body of evidence on the interaction between NCDs and COVID-19. About half of the HCWs had comorbidities at the time of admission, with the most common comorbidities being hypertension, diabetes mellitus, asthma, cardiac disease, and chronic pulmonary diseases (not asthma). A little over 50% of the HCWs suffered from severe COVID-19 during the period of hospitalization and roughly 11% died. Under the assumption of a causal relationship between the exposure and severity and mortality outcomes, we estimated that approximately 6.89% (95% CI, 4.65-9.22) of severe COVID-19 cases and 36.00% (95% CI, 28.8-44.5) of COVID-19-related deaths could potentially be averted if obesity and diabetes mellitus respectively were eliminated as risk factors.

Evidence from our study showed that COVID-19 affected more female HCWs than males in terms of hospitalization. Other studies have documented similar findings to ours by demonstrating female predominance of HCWs admitted for COVID-19. ^17^^,^^29^ A study conducted in Iran recorded a higher COVID-19 prevalence among female HCWs (53.5%) compared to males (46.5%), despite having a higher infection prevalence among males (56.4%) in the general population. ^29^ The reasons for more COVID-19 infection among female HCWs might be due to women being overrepresented in patient-facing healthcare roles such as nursing. Furthermore, women tend to have better health-seeking behaviour than men. ^30^ However, our finding has highlighted the need to explore more robust gender-focused IPC strategies to reduce this HCW vulnerability during infectious disease outbreaks.

Our study identified that about half (49.58%) of HCWs had at least one comorbidity at the time of admission. In keeping with our findings, other studies have also demonstrated the presence of at least one comorbidity and associated risk factors in half of the HCWs. ^25^^,^^31^ Furthermore, a 2021 systematic review examining the risk factors for SARS-CoV-2 infection among HCWs found an overall NCD prevalence of 18.4%; hypertension contributed the largest proportion (2.5%), followed by cardiovascular diseases (2.4%), chronic obstructive pulmonary disease (2.4%), and diabetes mellitus (1.4%). ^32^ However, this review did not examine an association between the NCDs and COVID-19 severity and mortality. It is clear that the burden of NCDs is high among HCWs as documented by the present available evidence including our study. NCD prevention and treatment should be prioritized among HCWs.

Evidence from our study indicated that there was an increased likelihood of the HCWs suffering from severe COVID-19 with older age (≥ 45 years), smoking, obesity, and being male. Additionally, we observed that asthma, cardiovascular diseases, and diabetes mellitus were positive predictors of COVID-19 severity. A 2021 study by Joo et al. documented an increased risk for severe COVID-19 in Malaysian HCWs with underlying comorbidities. ^33^

When HCWs are vulnerable to adverse health outcomes during an infectious disease outbreak it weakens the health systems’ response to and recovery from public health emergencies. This significant threat to the resilience of health systems highlights the imperative need for strategies to mitigate against the vulnerability of HCWs. ^34^

Specific risk factors were identified in our study that increased the predisposition for mortality from COVID-19 among the HCWs with COVID-19. These included being older than 45 years, male gender, obesity, and smoking. A WHO study aimed at estimating the impact of COVID-19 on HCWs also highlighted higher mortality (60%) among male HCWs. ^35^ Moreover, from a 2021 systematic review older age, male gender, and obesity heightened the risk of COVID-19 severity and mortality in the general population. ^36^ The study on all the COVID-19 patient data in the ISARIC database documented that obesity led to a 24% increased risk for mortality. ^37^ Looking at the increasing global burden of obesity, HCWs should embark on lifestyle modification activities that will avert the development of obesity.

Our study also found that cardiovascular disease, dementia, diabetes mellitus, chronic haematological disease, chronic kidney disease, chronic pulmonary diseases (not asthma), malignant neoplasm, chronic neurological disorder, and rheumatological disorders significantly increased the risk of COVID-19 deaths. This finding concurred with other studies which showed a higher mortality risk for COVID-19 patients with concurrent cardiac disease and diabetes mellitus. ^26^^,^^38^

In our analysis in terms of the PAF, obesity emerged as the leading risk factor for disease severity and diabetes mellitus as the leading risk factor for mortality associated with COVID-19. The PAF of 6.89% and 36.00% for severity and mortality respectively underscores the substantial impact that such modifiable factors could have on reducing COVID-19 severity and mortality rates. Similarly, a systematic review conducted in 2020 highlighted that the PAF for COVID-19 severity for obesity was 7.1% and that for diabetes mellitus within the general population was 6.5%. ^20^ By focusing on interventions aimed at reducing the incidence of obesity and diabetes mellitus, public health authorities can potentially achieve a dual benefit of mitigating the immediate impact of COVID-19 and enhancing the overall resilience of the HCWs against future infectious disease outbreaks and other health threats.

The findings from this study have several important implications. Due to the risks posed by NCDs to HCWs, we recommend the establishment of effective occupational health and NCD prevention, screening, and treatment programmes for HCWs. Investing in lifestyle interventions for the prevention of NCDs and associated risk factors particularly obesity and diabetes mellitus amongst HCWs to reduce NCD burden will generate dividends in reducing their vulnerability during infectious disease outbreaks. Additionally, based on the increased gender-related risk of COVID-19 among HCWs, health stakeholders need to explore gender-focused IPC advocacy among HCWs.

Our study had several strengths. First, we utilized a dataset collected from the largest cohort of hospitalized COVID-19 patients from 29 countries across 7 regions. This large sample size and the broad distribution of the HCW population give credence to the emerging evidence. This large and geographically broad sample also reduces biases that might be related to country-level data reporting which might underreport cases ^35^^,^^39^ and thus weaken the validity of the evidence. Second, we adhered to Strengthening the Reporting of Observational Studies in Epidemiology (STROBE) guidelines for reporting study findings. Third, we conducted a robust statistical analysis to generate the population-attributable fraction which will support the extrapolation of our study findings beyond our study population.

Despite the strengths, our study had some limitations. The majority of the patient information in the ISARIC database was from South Africa and the United Kingdom, whereas the other countries reported limited data. Since reporting to the ISARIC database was voluntary, the reasons for limited data from the other countries remain unknown. However, this underlying reason needs further exploration to develop solutions that will improve data sharing by countries and increase the robustness and geographic generalisability of the database. Based on this limitation, our findings should be generalized with caution. Second, the high number of missing values for some of the comorbidities limited the statistical power concerning some of the comorbidities.

## Conclusions

Our study revealed that many of the HCWs at the frontline of managing COVID-19 had NCDs which increased their vulnerability in the face of infectious disease outbreaks. One fifth of the admitted HCWs suffered from severe COVID-19 and one-tenth of HCWs died due to COVID-19. Additionally, HCWs who were ≥45 years old, male, smokers, and obese had a higher risk of increased severity and/or dying from COVID-19 disease. We recommend the implementation of health education and promotion activities to reduce the NCD burden among HCWs to reduce their vulnerability during infectious disease outbreaks.

## Data Availability

The data that underpin this analysis are available via a governed data access mechanism following a review of a data access committee. Data can be requested via the IDDO COVID-19 Data Sharing Platform ( http://www.iddo.org/covid-19). The Data Access Application, Terms of Access and details of the Data Access Committee are available on the website. Briefly, the requirements for access are a request from a qualified researcher working with a legal entity who have a health and/or research remit; a scientifically valid reason for data access which adheres to appropriate ethical principles, and has plans to promote equity in the use of data. The full terms are at: https://www.iddo.org/ebola/data-access-guidelines. These data are a part of https://doi.org/10.48688/cpwp-ft84. This article complied with the STROBE guidelines for reporting observational studies.

## References

[1] Ortiz DAP Abrigo MRM : The Triple Burden of Disease. *Economic Issue of the Day.* 2017; Vol.17. Reference Source

[2] Amuyunzu-Nyamongo M : Noncommunicable diseases, injuries, and mental health: the triple burden in Africa. *The Pan African medical journal. NLM (Medline).* 2022;43:167. 10.11604/pamj.2022.43.167.38392 PMC9941609 36825124

[3] Ladusingh L Mohanty SK Thangjam M : Triple burden of disease and out of pocket healthcare expenditure of women in India. *PLoS One.* 2018 May 1;13(5):e0196835. 10.1371/journal.pone.0196835 29746506 PMC5945049

[4] Rossati A : Global warming and its health impact. *International Journal of Occupational and Environmental Medicine.* 2017;8:7–20. NIOC Health Organization. 28051192 10.15171/ijoem.2017.963 PMC6679631

[5] Semenza JC Rocklöv J Ebi KL : Climate Change and Cascading Risks from Infectious Disease. *Infectious Diseases and Therapy. Adis.* 2022;11:1371–1390. 10.1007/s40121-022-00647-3 35585385 PMC9334478

[6] Leal J O’Grady HM Armstrong L : Patient and ward related risk factors in a multi-ward nosocomial outbreak of COVID-19: Outbreak investigation and matched case–control study. Antimicrob Resist. *Infection Control.* 2023 Dec 1;12(1). 10.1186/s13756-023-01215-1 PMC10031162 36949510

[7] Shah ASV Wood R Gribben C : Risk of hospital admission with coronavirus disease 2019 in healthcare workers and their households: Nationwide linkage cohort study. *The BMJ.* 2020 Oct 28;371. 10.1136/bmj.m3582 PMC7591828 33115726

[8] Nguyen LH Drew DA Graham MS : Risk of COVID-19 among front-line health-care workers and the general community: a prospective cohort study. *Lancet Public Health.* 2020 Sep 1;5(9):e475–e483. 10.1016/S2468-2667(20)30164-X 32745512 PMC7491202

[9] Abbas M Robalo Nunes T Martischang R : Nosocomial transmission and outbreaks of coronavirus disease 2019: the need to protect both patients and healthcare workers. *Antimicrobial Resistance and Infection Control.* 2021;10. BioMed Central Ltd. 10.1186/s13756-020-00875-7 PMC7787623 33407833

[10] Shaukat N Ali DM Razzak J : Physical and mental health impacts of COVID-19 on healthcare workers: A scoping review. *International Journal of Emergency Medicine.* 2020;13. BioMed Central Ltd. 10.1186/s12245-020-00299-5 PMC7370263 32689925

[11] Saifullah MZ Li M Maqbool MQ : Impact of COVID-19 pandemic on health care workers (HCWs) in Sindh Province of Pakistan. *Health Research Policy and Systems.* 2023 Dec 1;21(1). 10.1186/s12961-023-01022-5 PMC10388469 37525274

[12] Kamara IF Tengbe SM Fofanah BD : Infection Prevention and Control in Three Tertiary Healthcare Facilities in Freetown, Sierra Leone during the COVID-19 Pandemic: More Needs to Be Done!. *International Journal of Environmental Research and Public Health.* 2022 May 1;19(9). 10.3390/ijerph19095275 PMC9105082 35564669

[13] Ahmed J Malik F Bin Arif T : Availability of Personal Protective Equipment (PPE) Among US and Pakistani Doctors in COVID-19 Pandemic. *Cureus.* 2020 Jun 10;12(6):e8550. 10.7759/cureus.8550 32670687 PMC7357309

[14] Squire JS Dadzie D Nyarko KM : Risk Factors for COVID-19 infection among Hospital Healthcare Workers, Sierra Leone. 2020. 10.37432/jieph.supp.2022.5.1.04

[15] Tengbe SM Kamara IF Ali DB : Psychosocial impact of COVID-19 pandemic on front-line healthcare workers in Sierra Leone: an explorative qualitative study. *BMJ Open.* 2023 Aug 22;13(8):e068551. 10.1136/bmjopen-2022-068551 37607792 PMC10445370

[16] Dzinamarira T Murewanhema G Mhango M : COVID-19 prevalence among healthcare workers. A systematic review and meta-analysis. *International Journal of Environmental Research and Public Health.* 2022;19. MDPI. 10.3390/ijerph19010146 PMC8750782 35010412

[17] Bandyopadhyay S Baticulon RE Kadhum M : Infection and mortality of healthcare workers worldwide from COVID-19: A systematic review. *BMJ Global Health.* 2020;5:e003097. BMJ Publishing Group. 10.1136/bmjgh-2020-003097 33277297 PMC7722361

[18] Misra-Hebert AD Jehi L Ji X : Impact of the COVID-19 Pandemic on Healthcare Workers’ Risk of Infection and Outcomes in a Large, Integrated Health System. *The Journal of General Internal Medicine.* 2020 Nov 1;35(11):3293–3301. 10.1007/s11606-020-06171-9 32875500 PMC7462108

[19] El-Raey F Alboraie M Youssef N : Predictors for severity of sars-cov-2 infection among healthcare workers. *Journal of Multidisciplinary Healthcare.* 2021;14:2973–2981. 10.2147/JMDH.S335226 34729011 PMC8557804

[20] Li X Zhong X Wang Y : Clinical determinants of the severity of COVID-19: A systematic review and meta-analysis. *PLoS One.* 2021;16. Public Library of Science. 10.1371/journal.pone.0250602 PMC8092779 33939733

[21] Noncommunicable diseases WHO:[cited 2024 Mar 12]. Reference Source

[22] Gouda HN Charlson F Sorsdahl K : Burden of non-communicable diseases in sub-Saharan Africa, 1990–2017: results from the Global Burden of Disease Study 2017. *Lancet Global Health.* 2019 Oct 1;7(10):e1375–e1387. 10.1016/S2214-109X(19)30374-2 31537368

[23] Khargekar N Singh A Shruti T : A Cross Sectional Assessment of the Profile of Risk Factors of Non-Communicable Diseases Among Health Care Staff of a Tertiary Cancer Hospital. *Journal of Lifestyle Medicine.* 2022 May 31;12(2):98–103. 10.15280/jlm.2022.12.2.98 36157886 PMC9490011

[24] Domingues JG Silva BBC da Bierhals IO : Noncommunicable diseases among nursing professionals at a charitable hospital in Southern Brazil. *Epidemiologia e Servicos de Saude.* 2019;28(2). 10.5123/S1679-49742019000200011 31291438

[25] Calderwood CJ Marambire E Nzvere FP : Prevalence of chronic conditions and multimorbidity among healthcare workers in Zimbabwe: Results from a screening intervention. *PLOS Global Public Health.* 2024 Jan 23;4(1):e0002630. 10.1371/journal.pgph.0002630 38261562 PMC10805297

[26] Nikoloski Z Alqunaibet AM Alfawaz RA : Covid-19 and non-communicable diseases: evidence from a systematic literature review. *BMC Public Health.* 2021 Dec 1;21(1):1068. 10.1186/s12889-021-11116-w 34090396 PMC8178653

[27] Garcia-Gallo E Merson L Kennon K : ISARIC-COVID-19 dataset: A Prospective, Standardized, Global Dataset of Patients Hospitalized with COVID-19. *Science Data.* 2022 Jul 30;9(1). 10.1038/s41597-022-01534-9 PMC9339000 35908040

[28] Ferguson J O’connell M : graphPAF: An R package to estimate and display population attributable fractions.

[29] Sabetian G Moghadami M Hashemizadeh Fard Haghighi L : COVID-19 infection among healthcare workers: a cross-sectional study in southwest Iran. *Virology Journal.* 2021 Dec 1;18(1):58. 10.1186/s12985-021-01532-0 33731169 PMC7968574

[30] Thompson AE Anisimowicz Y Miedema B : The influence of gender and other patient characteristics on health care-seeking behaviour: A QUALICOPC study. *BMC Family Practice.* 2016;17(1):38. 10.1186/s12875-016-0440-0 27036116 PMC4815064

[31] Faruque M Barua L Banik PC : Prevalence of non-communicable disease risk factors among nurses and para-health professionals working at primary healthcare level of Bangladesh: A cross-sectional study. *BMJ Open.* 2021 Mar 19;11(3):e043298. 10.1136/bmjopen-2020-043298 33741665 PMC7986941

[32] Gholami M Fawad I Shadan S : The COVID-19 Pandemic and Health and Care Workers: Findings From a Systematic Review and Meta-Analysis (2020–2021). *International Journal of Public Health.* 2023;68. 10.3389/ijph.2023.1605421 36938301 PMC10020210

[33] Joo LK Sazali MF Goroh M : Predictors of severe COVID-19 among healthcare workers in Sabah, Malaysia. *BMC Health Services Research.* 2022 Dec 1;22(1):1541. 10.1186/s12913-022-08920-4 36528610 PMC9758662

[34] Okoroafor SC Asamani JA Kabego L : Preparing the health workforce for future public health emergencies in Africa. *BMJ Global Health.* 2022;7:e008327. BMJ Publishing Group. 10.1136/bmjgh-2021-008327 35414522 PMC9006823

[35] The World Health Organization: The impact of COVID-19 on health and care workers: a closer look at deaths. 2021.

[36] Li X Zhong X Wang Y : Clinical determinants of the severity of COVID-19: A systematic review and meta-analysis. *PLoS One.* 2021;16. Public Library of Science. 10.1371/journal.pone.0250602 PMC8092779 33939733

[37] Kartsonaki C Baillie JK Barrio NG : Characteristics and outcomes of an international cohort of 600 000 hospitalized patients with COVID-19. *International Journal of Epidemiology.* 2023 Apr 1;52(2):355–376. 10.1093/ije/dyad012 36850054 PMC10114094

[38] Pranata R Henrina J Raffaello WM : Diabetes and COVID-19: The past, the present, and the future. *Metabolism: Clinical and Experimental.* W.B. Saunders;2021;121. 10.1016/j.metabol.2021.154814 PMC8192264 34119537

[39] Gholami M Fawad I Shadan S : The COVID-19 Pandemic and Health and Care Workers: Findings From a Systematic Review and Meta-Analysis (2020–2021). *International Journal of Public Health.* 2023;68. 10.3389/ijph.2023.1605421 36938301 PMC10020210

