# Supplementary material for: The association between non-communicable diseases and COVID-19 severity and mortality among infected hospitalized healthcare workers in 29 countries: a cohort study
Source: F1000Res. 2025 Jun 11;13:624. Originally published 2024 Jun 12. [Version 2] doi: 10.12688/f1000research.150838.2 (PMC12274790; doi:10.12688/f1000research.150838.2)
Supplement: Peer review history

# Reviewer report: Reviewer response for version 2

**DOI:** 10.5256/f1000research.183478.r398522

**Published:** 2025-07-18

**Authors:** Bashir Bello

**Affiliations:** 1 Department of Sociology, Federal University, Gusau, Nigeria

The research is okay and indexable. The introduction was precise and concise and in it was a the statement of the problems. methodology is appropriate and  detailed. The data collected were properly analyzed and well presented. The subject matter was properly addressed.. The references also captured all what was presented. The research is okay just a brief grammatic editing

Is the work clearly and accurately presented and does it cite the current literature?

Yes

If applicable, is the statistical analysis and its interpretation appropriate?

Yes

Are all the source data underlying the results available to ensure full reproducibility?

Yes

Is the study design appropriate and is the work technically sound?

No

Are the conclusions drawn adequately supported by the results?

Yes

Are sufficient details of methods and analysis provided to allow replication by others?

Partly

Reviewer Expertise:

SOCIOLOGY, SOCIAL PROBLEM , INDUSTRIAL AND WORKS AND THEORY

I confirm that I have read this submission and believe that I have an appropriate level of expertise to confirm that it is of an acceptable scientific standard.

# Reviewer report: Reviewer response for version 2

**DOI:** 10.5256/f1000research.183478.r391878

**Published:** 2025-07-08

**Authors:** Recai Ergün

**Affiliations:** 1 University of Selcuk Faculty of Medicine, Konya, Konya, Turkey

The manuscript titled “Association between noncommunicable diseases and COVID-19 severity and mortality among infected hospitalized HCWs in 29 countries: a cohort study” presents an investigation into the role of noncommunicable diseases (NCDs) in COVID-19 outcomes among HCWs.

 This study includes a large multinational cohort of over 17,000 HCWs in 29 countries.

 The methodology is rigorous and uses appropriate statistical models (e.g., log-binomial regression) and population attributable fraction analysis to quantify the impact of various comorbidities.

 To enhance scientific transparency and rigor, the study follows STROBE reporting guidelines.

 The findings aim to contribute to public health by providing recommendations for occupational health strategies designed to protect HCWs during infectious disease outbreaks.

 The manuscript is well written, the data are clearly presented, and the conclusions are strongly supported by the results. I believe this study makes a valuable contribution to the literature on COVID-19 and HCW safety.

 I recommend that you accept the article in its current form.

Is the work clearly and accurately presented and does it cite the current literature?

Yes

If applicable, is the statistical analysis and its interpretation appropriate?

Yes

Are all the source data underlying the results available to ensure full reproducibility?

Yes

Is the study design appropriate and is the work technically sound?

Yes

Are the conclusions drawn adequately supported by the results?

Yes

Are sufficient details of methods and analysis provided to allow replication by others?

Yes

Reviewer Expertise:

Pulmonary vascular diseases and pulmonary hypertension; Occupational and environmental lung diseases; Interstitial lung diseases and fibrosis; Respiratory failure and mechanical ventilation; Critical care in respiratory medicine; Bronchoscopy and pleural procedures; COPD, asthma and obstructive lung diseases; Tuberculosis and post-tuberculosis lung disorders; Pulmonary infections and sepsis in ICU; Lung cancer and pulmonary nodules; COVID-19

I confirm that I have read this submission and believe that I have an appropriate level of expertise to confirm that it is of an acceptable scientific standard.

# Reviewer report: Reviewer response for version 1

**DOI:** 10.5256/f1000research.165440.r317489

**Published:** 2024-09-25

**Authors:** Max Carlos Ramírez-Soto

**Affiliations:** 1 Centro Nacional de Salud Puública, Universidad de San Martín de Porres, Lima, Peru

In this study they evaluate risk factors for COVID-19 in health care workers. Their findings reveal that age, sex, diabetes and cardiovascular disease are risk factors for severity and death. These risk factors have been studied extensively, in the general population and in different risk groups. Although the study has interesting data, further analysis is needed to prove the association described above. 

 Major comments:

 Methods.

 To check for compliance with the STROBE guideline items.

 Statistical analysis. What assumptions were used to calculate the RR. 

 Regarding the number of comorbidities, 2 or more should define multi-morbidity. 

 Results

 They should include a flow chart with information on included and excluded cases with their respective reasons. 

 Table 1 and 2. There are many unknowns, e.g., Current smoker, obesity, severity, Non-invasive ventilation, Inotropes, vasopressors, and High-flow oxygen nasal cannulas.  

 Table 3. The authors should revise their analyses. This table includes data from 7858 patients, however in the comparisons of variables, none of the sums of yes and no variables match.

 Table 4. They do not include ratios because they include the population 16674.

 The authors included the population between January 2020 and January 2023. Here, my main concern is that from 2021 onwards health workers were the first at-risk group to be vaccinated, but the authors did not include vaccination as an adjustment variable. Also, the study does not assess, nor does it compare variables before vaccination and after vaccination. It is likely that the highest number of cases occurred before vaccination (during 2020). For this year alone, a pre-vaccination analysis should be conducted to determine the association with risk factors, and another analysis in the vaccinated population (2021 to 2023), to assess whether the risk of age, sex, cardiovascular disease and diabetes persist in the vaccinated population compared to the unvaccinated. 

 Discussion and conclusions should be modified based on further analysis.

Is the work clearly and accurately presented and does it cite the current literature?

Yes

If applicable, is the statistical analysis and its interpretation appropriate?

Partly

Are all the source data underlying the results available to ensure full reproducibility?

Yes

Is the study design appropriate and is the work technically sound?

No

Are the conclusions drawn adequately supported by the results?

No

Are sufficient details of methods and analysis provided to allow replication by others?

No

Reviewer Expertise:

Infectious diseases

I confirm that I have read this submission and believe that I have an appropriate level of expertise to confirm that it is of an acceptable scientific standard, however I have significant reservations, as outlined above.
